# Mobile Phone Access, Usage Patterns, and Perceptions of Adolescents Living With HIV on the Use of Gamified Interventions to Improve Antiretroviral Therapy Adherence in Eswatini: Qualitative Study

**DOI:** 10.2196/74207

**Published:** 2026-07-24

**Authors:** Londiwe D Hlophe, Peter S Nyasulu, Constance S Shumba

**Affiliations:** 1 Division of Epidemiology and Biostatistics, Faculty of Medicine and Health Sciences, Stellenbosch University, PO Box 241, Cape Town, 8000, South Africa, 268 76135612; 2 Division of Epidemiology and Biostatistics, Faculty of Health Sciences, University of the Witwatersrand, Johannesburg, South Africa; 3 Division of Epidemiology and Social Sciences, Institute for Health and Equity, Medical College of Wisconsin, Wisconsin, United States

**Keywords:** adolescents living with HIV, antiretroviral therapy, ART adherence, mobile phones, mobile health, gamified interventions, Eswatini

## Abstract

**Background:**

Adolescents living with HIV often experience poor antiretroviral therapy (ART) outcomes due to multiple barriers affecting medication adherence. Effective self-care interventions are needed to address these challenges. Mobile phones are widely used by the adolescent population and therefore present an opportunity to enhance ART adherence using mobile phone–based interventions. However, research on mobile phone access among adolescents living with HIV, usage patterns, and perceptions of mobile phone–based interventions is limited in Eswatini.

**Objective:**

This study aimed to explore these aspects to inform effective mobile health strategies for enhancing ART adherence among adolescents living with HIV.

**Methods:**

We conducted a qualitative study using in-depth interviews in December 2023. A total of 29 adolescents living with HIV aged 10 to 19 years and enrolled on ART were purposively sampled and interviewed from 5 Teen Clubs in the Hhohho region of Eswatini. Interviews were audio-recorded and transcribed verbatim. Topic areas covered were mobile phone accessibility, usage patterns, and perceptions on the use of mobile phones to facilitate ART adherence. The data were analyzed using the deductive-inductive coding approach.

**Results:**

Of the 29 participants, 15 (52%) were female, and 19 (65.5%) were aged between 15 and 19 years. The study findings indicated high mobile phone access among participants, with primary usage focused on making and receiving calls, as well as engaging with social media. Three themes emerged regarding the use of gamified interventions to support ART adherence. First, the use of gamified interventions aimed at ART adherence among adolescents living with HIV was deemed feasible based on mobile phone access and past experiences with mobile games. Second, 3 main qualities of successful gamified interventions were identified as being supportive, being educational, and ensuring secure and confidential connections with other players. Finally, confidentiality and mobile phone access factors were highlighted as potential concerns when designing gamified ART adherence interventions.

**Conclusions:**

The findings suggest potentially high access and usage of mobile phones among adolescents living with HIV on ART in Eswatini. This provides an opportunity to leverage mobile technology to enhance ART adherence through gamified interventions. However, it is essential to carefully consider the specific needs and concerns of adolescents living with HIV in the design of these interventions to ensure their successful uptake and sustainability.

## Introduction

Eswatini’s adolescents have consistently shown lower antiretroviral therapy (ART) outcomes, such as viral load suppression (VLS), compared to the adult population. In 2023, Eswatini recorded a 96% VLS rate among adults, while adolescents aged 15 to 19 years reported a 77.8% viral suppression rate among those on ART [1,2]. Although there has been a very slight improvement compared to 2017 data where VLS was 76% among adolescents and young people, it remains below the Joint United Nations Programme on HIV/AIDS (UNAIDS) 90% VLS target for 2020 [3,4]. Low VLS is associated with poor ART adherence, leading to opportunistic infections and subsequently AIDS-related deaths [5-7].

Nonetheless, Eswatini has made tremendous progress in combating the HIV epidemic, despite having the highest prevalence of HIV globally at 24.8% [1]. This progress has positioned the country among the first to meet the 2030 UNAIDS targets ahead of the set date [1,2]. The UNAIDS targets aim for 95% of people living with HIV to know their status, 95% of those diagnosed to be on ART, and 95% of those on ART to achieve viral suppression by 2030 [8]. Eswatini has achieved these targets through successfully implemented interventions promoting ART adherence [2]. For instance, in 2017, the country adopted the universal test and treat guidelines and coupled them with interventions promoting ART adherence counseling, resulting in the implementation of a differential model of ART care [9].

However, globally, interventions aimed at improving ART adherence have proven to be more effective in the adult population, while adolescents remain behind [10]. A systematic review conducted in 2015 highlighted that few interventions have been implemented among younger population groups globally [10]. This discrepancy is particularly pronounced in sub-Saharan Africa (SSA), which is home to 70% of the global population living with HIV and where adolescents account for 90% of the global adolescents living with HIV [8,11]. ART outcomes are also poor among the adolescent age group, resulting in 65% ART adherence and 55% VLS among adolescents in the region [12]. Despite these challenges, interventions targeting ART adherence among adolescents living with HIV are limited compared to those available for adults [13,14]. Only a few interventions, such as HIV counseling, community and facility-based support, and family-based economic empowerment programs, have been reported in previously conducted studies [10,15-17].

The potential of digital technology, particularly mobile health (mHealth), in supporting ART adherence among adolescents living with HIV remains largely underexplored, as most studies continue to combine adolescents with young adults [18,19]. For example, a systematic review of mHealth interventions targeting adolescents and young adults identified 8 studies focused on ART adherence in this population [19]. Of these, only 1 study exclusively targeted adolescents; the rest grouped adolescents with young adults, and 1 study even included children [19]. Furthermore, studies have been conducted in only 5 countries within SSA (Nigeria, Kenya, Uganda, South Africa, and Zimbabwe), highlighting the still limited implementation and evaluation of mHealth-based interventions in other high-burden settings such as Eswatini, which has the highest HIV prevalence globally [14,19]. Despite this limited focus, evidence from other African countries indicates that mHealth interventions can significantly improve adherence rates and VLS among young populations [19,20]. The positive influence of mobile phone–based interventions has been bolstered by the widespread accessibility and usage of phones among young people. As of 2024, an estimated 5.4 billion individuals owned mobile phones globally, with a total of 9.1 billion mobile phone subscriptions registered worldwide [21]. On the African continent, approximately 577 million people owned mobile phones, accounting for 1.1 billion mobile subscriptions [21]. Regarding internet access, 5.5 billion people (68% of the global population) were internet users in 2024, including 456 million individuals in Africa [21]. Notably, young people represent the most connected demographic, with 78.5% of global internet users aged between 15 and 24 years. In Africa, young people constituted 53% of internet users [21]. In 2022, Eswatini, a country with 1.3 million people, reported 1.5 million phone subscriptions [22]. Although specific data on phone ownership among adolescents are scant, a systematic review showed that young people and adolescents spend a significant amount of time on their phones, leading to phone addictions [23]. Adolescents have been reported to be particularly addicted to social media and gaming [24,25]. Studies have also reported the positive effect of mobile games in promoting medication adherence [26]. However, there is a need for tailored interventions where the end user actively participates in the design of these interventions [27].

Therefore, access to phones can be leveraged in settings such as Eswatini, where adolescents have poor ART outcomes compared to the adult population. However, there are limited data on phone ownership, accessibility, and usage among adolescents in the country. Additionally, studies have shown the importance of end-user participation in the design of health behavior interventions. We explored mobile phone access, usage patterns, and perceptions of adolescents living with HIV regarding the use of gamified interventions aimed to improve ART adherence in Eswatini.

## Methods

### Study Design

This was a qualitative study conducted in December 2023 using in-depth interviews (IDIs) with 29 adolescents living with HIV in Eswatini. This approach provided valuable insights from adolescents living with HIV, who have a unique understanding of the types of interventions that may be most effective for them [28].

### Study Setting

The study was conducted among adolescents living with HIV accessing ART care through Teen Clubs in the Hhohho region in Eswatini. The Hhohho region is located in the northwestern part of the country and houses the capital city, accounting for 30% of the total population [29]. This region is considered the most economically advanced in Eswatini, as it has the most urbanized population and is home to the headquarters of many corporations, as well as being dominated by the forestry industry, which is among the most important sectors of the country’s economy [29]. The region comprises both urban and rural settings and has 71 health facilities, including private, public, military, faith-based, and not-for-profit hospitals, health centers, public health units, and clinics [30]. These health facilities play a critical role in HIV prevention and management through services offered from the community level to the tertiary level (hospitals). These services include the differentiated models of ART care, such as Teen Clubs [9].

Teen Clubs, a model of ART care for adolescents with disclosed HIV status, use lay health care workers also known as expert clients to provide support and education about ART and HIV [9,31]. Expert clients are HIV-positive adults whose primary role includes providing adherence counseling, tracking clients lost to follow-up, conducting pill counts to monitor medication adherence, and supporting adolescents in navigating psychosocial challenges such as stigma, suicidal ideation, and orphanhood [31]. They work collaboratively with nurses to help alleviate the clinical workload involved in managing adolescents living with HIV [31]. In 2023, there were 99 Teen Clubs with 2308 members in the country (Swaziland National AIDS Programme, National Facility Teen Clubs, Eswatini Ministry of Health, unpublished report, 2023). Of these, 31 Clubs with 1464 members were from the Hhohho region. With the assistance of the Ministry of Health, Swaziland National AIDS Program (SNAP), and the National Paediatrics HIV Care and Treatment department, 5 clubs from the Hhohho region that had been active for at least 2 years were conveniently chosen for data collection [28]. Within each club, at least 5 adolescents living with HIV meeting the inclusion criteria were purposively selected [32]. To ensure the representativeness of perceptions and experiences of all adolescents living with HIV in Teen Clubs, the selected participants included both male and female participants, adolescents from both age groups (10-14 years and 15-19 years), and at least 1 peer educator from each club [32]. Peer educators are also known as community adherence to treatment support (CATS) and are involved in educating and supporting their peers using door-to-door campaigns, especially for adolescents living with HIV lost to follow-up or with poor ART outcomes [33]. The inclusion criteria required adolescents aged 10 to 19 years who were aware of their HIV status and had been members of the Teen Club for at least 6 months at the time of data collection to ensure a comprehensive ART history.

### Participant Recruitment Process

Expert clients assisted in the identification of potential participants and were referred to the researcher for further explanation of the study. Adolescents who were willing to be part of the study and met the inclusion criteria were given a detailed study information sheet in both siSwati and English. Adolescents aged below 18 years were given the information sheet, along with the researcher’s contact information, consent, and assent forms to share with their guardians. Adolescents aged 18 years provided their voluntary consent to participate in the study. Interviews took place in December 2023 following the receipt of signed consent and assent forms. A total of 29 adolescents meeting our inclusion criteria were recruited. The sample size was guided by the principle of data saturation in conducting qualitative studies [32]. The sample size for achieving data saturation was informed by previous studies conducted among similar populations, using comparable data collection tools, specifically semistructured interview guides [34,35]. Data saturation was achieved when no new relevant information emerged from the participants [28]. Data saturation was assessed by evaluating the repetitiveness of responses. Specifically, data collection continued until no new or significant information emerged from the interviews.

### Data Collection and Management

An IDI guide was developed based on an extensive literature review of studies examining barriers and facilitators of ART adherence, as well as interventions targeting ART adherence among adolescents living with HIV. The interview guide included topics on ART and ART adherence experience, mobile phone accessibility and usage patterns, barriers and facilitators to ART adherence, and perceptions of the use of mobile phones to facilitate ART adherence (Multimedia Appendix 1). To ensure the validity of the questions, the interview guide was pilot-tested through 2 interviews with adolescents living with HIV who were not included in the final sample [28]. The interview guide, along with all consent and assent supporting materials, was translated into siSwati to ensure comprehension by all participants. Consequently, the IDIs were conducted in either siSwati or English, depending on the participants’ preferences, and were audio-recorded. Each interview lasted about 45 minutes, and interviews were conducted in private rooms provided by each health facility or other locations of participants’ preference. To protect participants’ identities and ensure confidentiality, personal identifiers, such as names, were not used during interviews.

The audio recordings were transcribed verbatim, and those in siSwati were translated into English. Personal identifiers of participants were not included in both the transcripts and audio files; instead, codes were used to identify participants to maintain confidentiality. Both the transcripts and audio recordings were securely stored on a password-protected computer.

### Data Analysis

A deductive-inductive coding approach was used for data analysis [36]. The initial code frames were developed based on the research question being “Can mobile games be used to facilitate ART adherence?”, while additional themes emerged inductively from the participants’ responses. A code framework was developed, and transcripts were coded manually [28,32]. The code book was reviewed by 2 independent investigators (LDH and CSS) for comprehensiveness and appropriateness [37]. Differences were discussed and resolved collaboratively by the 2 investigators (LDH and CSS), and the final codebook was reviewed and validated by PSN.

### Ethical Considerations

Approval to conduct the study was sought from the Stellenbosch University Health Research Ethics Committee (reference number: S24/02/037) and Eswatini Health and Human Research Review Board of the Ministry of Health (protocol reference number: EHHRRB143/2023). Furthermore, a letter of support from hospital management teams was obtained from the Ministry of Health, Department of Health Services. Finally, all participants and guardians in case of participants below the age of 18 were given detailed information on the study and provided consent and assent. Participants were informed that participation in the study was voluntary, and they were free to withdraw from the study at any point.

## Results

### Demographic Characteristics of Participants

A total of 29 participants (n=14 males and n=15 females) were included in the study. The majority (n=19, 65.5%) of the participants were aged 15 and above. The mean age of the participants was 16.5 (SD 1.08) years, with an average of 10.8 (SD 3.99) years on ART and 5 (SD 3.99) years of Club membership. The majority (n=23, 79%) of the participants were diagnosed with HIV from birth, while only 2 (7%) were diagnosed later in life (Table 1).

**Table 1.** Characteristics of participants.

Characteristics	Values, n (%)
Age group (y)	
10‐14	10 (34.5)
15‐19	19 (65.5)
Sex	
Female	15 (51.7)
Male	14 (48.3)
Education level	
Primary	9 (31.0)
High school	18 (62.1)
Tertiary	2 (6.9)
Age at diagnosis	
From birth	23 (79.3)
While young	4 (13.8)
Later in life	2 (6.9)
Duration on ART^a^ (y)	
<5	2 (6.9)
5-10	15 (51.7)
>10	12 (41.4)
Duration in club (y)	
<2	6 (20.7)
2-5	12 (41.4)
>5	11 (37.9)

a ART: antiretroviral therapy.

### Phone Ownership and Usage

A total of 28 (96.6%) participants either owned (n=19, 65.5%) or shared (n=9, 31%) a phone with their guardian or siblings. Among those who owned or had access to a phone, 16 (57.1%) used a smartphone, while 12 (42.9%) used a feature phone (Table 2). The majority (17/19; 89.5%) of those who owned phones were older adolescents (15-19 years old), and younger adolescents (7/9; 77.8%) were most likely to share the phone with either guardian or siblings. Only older adolescents reported uncontrolled time on the phone, while younger adolescents had their phone usage controlled by guardians.

**Table 2.** Phone ownership and usage characteristics of participants.

Characteristics	Age 10-14 years, n (%)	Age 15-19 years, n (%)	Total, n (%)
Phone ownership			
Yes	2 (20.0)	17 (89.5)	19 (65.5)
No	1 (10.0)	0 (0)	1 (3.4)
Shared	7 (70.0)	2 (10.5)	9 (31.0)
Type of phone			
Smartphone	4 (40)	12 (63.2)	16 (55.2)
Feature or basic phone	5 (50)	7 (36.8)	12 (41.4)
N/A^a^	1 (10)	0 (0)	1 (3.4)
Phone usage^b^			
Daily	6 (66.7)	19 (100)	25 (89.3)
Controlled time	3 (33.3)	0 (0)	3 (10.7)
Time spent on phone^b^			
Uncontrolled time	0 (0)	13 (68.4)	13 (46.4)
<1 hour per day	5 (55.6)	2 (10.5)	7 (25.0)
1 hour to 5 hours daily	1 (11.1)	4 (21.1)	5 (17.9)
Only on weekends and school holidays	3 (33.3)	0 (0)	3 (10.7)
Activities^b^			
Calling and receiving	9 (100)	19 (100)	28 (100)
Social media (WhatsApp, TikTok, Instagram, Facebook)	3 (33.3)	12 (63.2)	15 (53.6)
Games	2 (22.2)	5 (26.3)	7 (25.0)
Researching	1 (11.1)	2 (10.5)	3 (10.7)

a N/A: not applicable.

b Number of participants with phone access.

### Themes on the Perceptions of Gamified Interventions Among Adolescents Living With HIV

#### Overview

Three themes reflecting the perceptions of adolescents living with HIV on the use of gamified interventions to improve ART adherence emerged in this study. These were (1) feasibility of gamified interventions, (2) qualities of gamified interventions, and (3) concerns about the use of gamified interventions to enhance ART adherence (Table 3). Detailed themes with the supporting codes are provided in Multimedia Appendix 2.

**Table 3.** Themes, subthemes, and codes on the use of gamified interventions to enhance antiretroviral therapy (ART) adherence among adolescents living with HIV.

Themes and subthemes	Code
Gamified interventions are feasible	
Phone access and use	Previous game exposure Positive game experience
Qualities of gamified interventions	
Supportive	Reminders Support group
Educational	Benefits of ART Living with HIV
Secure and confidential connection	Dependable or trustworthy Prevention of unintended disclosure
Potential concerns about gamified interventions	
Confidentiality	Unintended disclosure
Phone access factors	Phone costs Internet bundles cost Network availability

#### Gamified Intervention Feasibility

Most participants reported that the use of gamified interventions to support ART adherence among adolescents living with HIV was feasible, given their access to mobile phones and previous exposure to games. Thus, participants reported that the success of gamified interventions is not only based on mobile phone access but also on having prior experience of playing other mobile games:

*You see auntie, we play lots of games so this can work too but then like myself I can only play it once in a while you see because I don’t own the phone … but it can help.*

[Female adolescent living with HIV, aged 15]

Additionally, the practicality of a gamified intervention was perceived to be influenced by the participants’ experiences with games, particularly when those experiences were positive. A participant also further noted that a gamified intervention might be more appealing to younger adolescents than older adolescents:

*I think it can work auntie especially among the young ones because those ones enjoy playing games… myself, I am not a game person but into blogs and other social media platforms because I don’t find games interesting you see.*

[Male adolescent living with HIV and peer educator, aged 19]

#### Qualities of Gamified Intervention

##### Overview

Participants reported that the gamified interventions should possess certain qualities for them to yield positive results in improving ART adherence among adolescents living with HIV. The qualities of a gamified intervention included being able to provide support to the players, being educational, and finally ensuring user HIV status privacy.

##### Supportive

First, most participants reported that a game aimed at improving ART adherence should offer support to the players, as they identified support as a key facilitator for ART adherence:

*You see auntie, some of us will benefit from a mobile game if it provides support as it’s hard to live with HIV especially where everyone else is negative … It will be like you have something you can do and like someone who understands you and when you play you are happy.*

[Male adolescent living with HIV, aged 18]

A mobile game that acts as a reminder for taking their medication would be very vital, as participants indicated that they often forget to take their medication on time:

*You see auntie, a game where like when you play you learn in the process. Like when you play you can even remember to take your pill and know why it is important.*

[Female adolescent living with HIV, aged 18]

Finally, some participants revealed that a multiplayer game could provide support through networking and sharing of experiences, especially if the game can be played in real-time with others:

*You know a game where I can be in my home and play with someone who is in Manzini, that one can work and even better if I know the person is also living with HIV and we can even chat outside the game even though we don’t know each other … sometimes talking to a stranger is better auntie because they won’t gossip about you.*

[Male adolescent living with HIV, aged 19]

*You know those racing games auntie where you have more than one player and even better if you know the players are real … those games are the best.*

[Male adolescent living with HIV, aged 17]

##### Educational

Participants reported that a mobile game with an educational component could benefit adolescents living with HIV on ART and improve adherence. They explained that the educational benefits would include information on the advantages of ART and guidance on living with HIV. This information would be critical in teaching players the importance of ART adherence and encouraging them to persevere despite life distractions:

*For me a game where you are fighting using a gun and when you shoot it’s an indication that what happens or the results of taking your pills.*

[Female adolescent living with HIV, aged 17]

*[T]he game can work for us like encourage us while teaching us about the pills That’s what I can say … it can be a game like avatar you see where if you fall at this point, even your life falls … yes auntie like reminds us that even if you forget your pill, don’t give up like the game when they shoot you lose a life but when you keep fighting you gain more lives, something like that.*

[Male adolescent living with HIV, aged 19]

Additionally, an educational game could be crucial in guiding adolescents living with HIV as they make life choices that influence their future. These choices include staying positive about life and teaching others that living with HIV does not mean a shortened lifespan; one can still live and enjoy their life:

*I think a game that will teach people when they play about life with HIV so that they know more and the things to do to make sure one stays well.*

[Female adolescent living with HIV, aged 14]

*[S]ometimes you have questions about HIV in general and you have to wait for the day you go to the health facility, you see the game can be helpful if it can answer some of the questions and you can even play with other people like children so they know more about HIV because now it’s like HIV means one will die … like I use poetry to teach my friends and imagine if it’s a game and we play and they learn.*

[Female adolescent living with HIV, aged 17]

##### Secure and Confidential Connection

Participants emphasized the importance of privacy and confidentiality in mobile games, especially those incorporating social connection features. While they expressed interest in multiplayer games, they strongly preferred designs that would safeguard their privacy and prevent unintended disclosure of their HIV status during gameplay. Participants indicated a preference for playing with strangers, believing this would better protect their identity. However, if the game allowed communication between players, they suggested it would be more acceptable if the other players were also living with HIV. This would create a safe space for sharing experiences without fear of stigma, gossip, or unwanted disclosure:

*You know a game where I can be in my home and play with someone who is in Manzini, that one can work and even better if I know the person is also living with HIV and we can even chat outside the game even though we don’t know each other … sometimes talking to a stranger is better auntie because they won’t gossip about you.*

[Male adolescent living with HIV, aged 19]

### Potential Concerns About the Gamified Interventions

#### Overview

Participants reported concerns regarding a mobile game aimed at supporting ART adherence among adolescents living with HIV. These concerns were critical to consider in the design of the intervention to ensure its success. Participants highlighted 2 main concerns: the possibility of the intervention leading to unintended disclosure of their HIV status and factors associated with phone access and use, which include the financial implications and network access.

#### Unintended Disclosure

Most participants revealed that inasmuch as they believed a mobile game can assist in supporting ART adherence, there were concerns regarding unintended disclosure if the name or some of the information could be traced to HIV or people living with HIV:

*We play games like me I normally play games from my mom’s phone, and I enjoy it a lot (laughs) but then auntie what if someone sees the game and can tell that it’s about HIV and now, they will be talking about my status.*

[Female adolescent living with HIV, aged 17]

*A game one I can play and even if someone can see but will not be able to tell it’s for people living with HIV … like my cousins can also play it without them knowing it’s for us.*

[Female adolescent living with HIV, aged 14]

#### Mobile Phone Access Factors

Mobile phone access was identified by participants as a key concern in the potential success of gamified interventions. Specifically, participants raised concerns about smartphone ownership, noting that while they had access to basic or feature phones, they did not own smartphones, which are typically required to run gamified apps:

*Mobile games can work auntie, but problem is like myself, I don’t have a phone and the one I use is not a smart phone so this will not benefit some of us.*

[Female adolescent living with HIV, aged 15]

Second, participants expressed concerns that even if the games could work, the cost of internet bundles would deter their success. Most adolescents do not have income and would be unable to afford the cost of internet or data bundles that would be required for online games:

*You see auntie, some of the adolescents here cannot afford to buy internet bundles and you know some games need you to be online.*

[Male adolescent living with HIV and peer educator, aged 19]

Finally, participants cited limited mobile network coverage in some of the geographical locations in the country as making it difficult to have access to a mobile phone. They believed that the lack of mobile network coverage would hinder some of them from taking part in the games:

*I don’t know auntie, but you know there are places where there is no network … (laughs) you see at my granny’s place, there is no network it’s just some odd area right (laughs).*

[Female adolescent living with HIV, aged 18].

## Discussion

### Principal Findings

The study findings suggest that mobile phone access among adolescents living with HIV in Eswatini may be high, although this was a small qualitative sample, providing a potentially solid foundation for the implementation of mHealth interventions targeting this age group. Currently, mHealth use in the country is largely limited to calls and SMS text message reminders for clinical appointments sent by health care workers [9]. There may be opportunities to expand the scope of mobile phone–based interventions among adolescents.

Older adolescents reported owning smartphones, while younger adolescents often shared phones with siblings or caregivers. This suggests the need for tailored age-appropriate mHealth solutions, with younger adolescents requiring simpler, lower-bandwidth tools compatible with feature phones and shorter usage durations due to parental control, while older adolescents could benefit from more interactive, internet-based interventions as observed in a study conducted in Zimbabwe [38]. However, while smartphone ownership is critical for implementing internet-based mHealth solutions, such interventions require stable internet connectivity, which may be hindered by limited coverage, slow internet speeds, and high costs [38]. This is particularly important in Eswatini, where network and internet access remain limited in some places in the country [39]. As of 2024, only 50.3% of the population had internet access, and the cost of 1 GB of mobile data was US $5.82 [39]. Therefore, given that 58.9% of the population lives below US $3.65 per person per day, mHealth interventions requiring regular internet use should be implemented cautiously to avoid excluding lower-income users [40].

Additionally, phone usage patterns varied by age. All adolescents used phones primarily for calls, while older adolescents were more likely to engage with social media platforms too. This underscores the importance of designing mHealth interventions with adaptable features that align with adolescents’ communication habits and preferences [41]. For instance, a study in Uganda found that adolescents preferred not only voice calls but also SMS text messaging and WhatsApp messaging, highlighting the potential value of integrating social connectivity features into mHealth interventions [41]. However, such features should be used with caution in Eswatini due to the cost of mobile services. As of 2024, calls cost approximately US $0.07 per minute, while an SMS text message cost US $0.02 in Eswatini [39].

The study further identified 3 core themes: feasibility, desired qualities, and potential concerns, which are vital to informing the design of gamified interventions. These themes offer valuable insights into the practicality and acceptability of such interventions.

In terms of feasibility, participants expressed confidence in the potential of mHealth, particularly mobile games, to improve ART adherence, despite only a quarter having prior gaming experience. This highlights the importance of considering players’ prior experience in the design of gamified interventions. According to Fitzgerald et al [42], games that promote user engagement are typically those that are easy to play, intuitive, and do not require previous gaming skills or knowledge [42]. This is supported by Sardi et al [43], who emphasized that effective games should be adaptive, offering various modes of play to accommodate different player types and skill levels [43]. Consequently, involving users in the design process is critical to ensure that the resulting intervention is user-centered and tailored to their needs, preferences, and abilities [43]. Additionally, the selection of appropriate gaming elements or mechanics is crucial, as these features can significantly influence user engagement and motivation. For instance, storytelling or narrative elements help set clear expectations and create an immersive experience that puts players at ease, enhancing their willingness to engage with the game [44]. Other mechanics, such as onboarding tutorials, rewards, and leaderboards, have been shown to promote sustained engagement by guiding new players and offering in-game incentives that reinforce continued participation [44,45]. Despite participants’ limited prior exposure to gaming, existing evidence supports the effectiveness of gamified mHealth interventions in improving medication adherence among adolescents [19]. These findings highlight the feasibility and potential impact of well-designed, user-centered gamified tools in supporting ART adherence among adolescents living with HIV.

Regarding the desired features or qualities of the gamified intervention, the study highlighted the importance of aligning game content and features with the preferences and needs of end users. First, participants emphasized that effective gamified interventions must include relevant, accurate, and engaging educational content, particularly on HIV and the benefits of ART. This finding aligns with a study conducted among adolescents living with HIV in the United States, where the integration of health education into games improved knowledge retention and supported positive behavior change while maintaining user engagement [46].

Second, the study revealed that incorporating a strong support system within the intervention could enhance its effectiveness in promoting ART adherence. Participants identified support in the form of medication reminders and opportunities for networking with other adolescents as essential. Literature consistently highlights the value of such support in improving ART adherence among adolescents living with HIV [47,48]. In a gamified context, support can be delivered through features such as in-game pop-up messages reminding players of important health behaviors or through social connectivity that allows users to share progress and communicate with peers. Platforms such as social media can offer avenues for users to exchange experiences, provide encouragement, and reinforce positive behaviors. However, the inclusion of social features must be approached with caution. Participants expressed concern that social connectivity within the game could compromise their privacy and lead to unintended disclosure of their HIV status. This concern is echoed in broader literature, where privacy and confidentiality have been flagged as critical risks in mHealth interventions that incorporate social connection and collaboration features [45,49]. Although social connectivity ranks as the second most frequently used game element, it raises substantial concerns about exposing players’ identities, particularly among vulnerable populations such as adolescents living with HIV [45]. These findings emphasize the need to design stigma-sensitive and developmentally appropriate interventions [49]. Adolescence is a period marked by emotional and cognitive maturation, yet formal reasoning and risk assessment abilities may still be developing [50].

Therefore, developers must carefully evaluate whether and how to include socially driven elements. Recommended approaches include the use of anonymous avatars and nicknames, while disabling features such as voice and video calls, and restricting media sharing strategies successfully implemented in the design of the PEERNaija game [51]. In the context of Eswatini, the findings suggest that integrating gamified mHealth interventions into existing service delivery structures, such as Teen Clubs, could enhance both effectiveness and user safety. Teen Clubs are trusted, adolescent-friendly platforms that provide safe spaces for sharing experiences and peer support without compromising privacy [52]. Leveraging peer educators within these settings could further enhance the intervention’s reach and acceptability [52].

Finally, regarding potential concerns, the study highlighted the critical importance of privacy and confidentiality in the design of gamified interventions. Participants expressed concerns about the risk of unintended disclosure, particularly among younger adolescents who often share mobile phones with siblings or caregivers. Such unintended disclosure could lead to stigma and discrimination, which are well-documented barriers to ART adherence among adolescents living with HIV [53]. Therefore, the game’s design, including its name, features, and interface, must be carefully considered to prevent any breach of confidentiality that could negatively affect adolescents’ willingness to engage with the intervention [54].

In addition to privacy concerns, participants raised issues related to the financial burden of mobile gaming and limited access to network services. These concerns underscore the need to critically evaluate the economic feasibility of mHealth interventions in low-resource settings. Adolescents must not only own a mobile phone, ideally a smartphone, but also maintain consistent access to mobile data and internet services. This requirement presents a significant challenge in contexts such as Eswatini, where economic constraints and infrastructural barriers, such as poor network coverage, limited electricity, and low digital literacy, can impede the successful implementation of mHealth solutions [38]. Similar challenges have been observed in other low-income settings, where the high cost of smartphones and internet bundles has contributed to poor uptake and limited effectiveness of mHealth-based interventions [38,41,55]. To mitigate these barriers, recent innovations in offline mobile gaming have shown promise. Offline games eliminate the need for constant internet access, thereby reducing the financial burden on users and improving accessibility. Studies have shown that offline interventions are associated with higher uptake and more favorable outcomes, particularly in resource-limited environments [56]. As such, the development of offline gamified interventions could represent a practical and sustainable solution to improve ART adherence among adolescents living with HIV in Eswatini and similar contexts [57].

Nevertheless, for mHealth interventions to be applicable, engaging, feasible, and effective, they should be firmly grounded in behavior change theories [58]. These theories provide a comprehensive framework for understanding adolescent behavior, particularly during the adolescence stage, a developmental period characterized by significant psychological, social, and physiological changes that can influence ART adherence [50,59]. By applying these theories, designers can identify key determinants of adherence and gain valuable insights into how behavior change can be effectively promoted [50]. Behavior change theories also inform the selection of appropriate game elements and design features that resonate with adolescents’ needs and preferences, thereby ensuring that user concerns are addressed during the design process [58]. Furthermore, they guide the integration of behavior change techniques that can enhance the intervention’s overall impact on ART adherence [50,58,59].

Critically, involving adolescents in the design process is essential. Their participation enables the cocreation of game features that directly reflect their lived experiences, needs, and expectations, thereby increasing the likelihood of sustained engagement and intervention effectiveness [59]. Health behavior models, such as social cognitive theory, the information-motivation-behavioral (IMB) skills model, and motivational interviewing, have been widely applied in mHealth interventions targeting adolescents [50]. These models conceptualize ART adherence as a behavior influenced by a combination of accurate information, individual and social motivation, and the acquisition of behavioral skills. When applied effectively, they enhance adolescents’ self-efficacy and lead to improved ART adherence outcomes [50]. Thus, grounding intervention design in these theories, developers can ensure that components are aligned with adolescents’ realities and are both contextually relevant and capable of promoting sustainable behavior change [50,58].

### Limitations and Strengths

A key limitation of this study is that it only included adolescents from one region of the country, specifically those who are members of Teen Clubs. Additionally, the small sample size is not representative of all adolescents living with HIV in the country or even within the Hhohho region. As a result, the findings cannot be generalized to all adolescents in Eswatini. Despite these limitations, this study is the first to explore and describe mobile phone access, usage patterns, and perspectives on gamified interventions among Swati adolescents living with HIV. The findings provide valuable baseline data and serve as a foundation for future research aimed at developing and evaluating gamified interventions to enhance ART adherence among adolescents living with HIV.

### Conclusion

The study indicates that mobile phone access may be high among adolescents living with HIV in Eswatini, with the majority using their phones daily for various purposes. A considerable proportion of participants expressed a belief in the feasibility of gamified interventions to support ART adherence, highlighting the importance of supportive, educational, and privacy-assured features. Nonetheless, concerns related to confidentiality and economic barriers must be addressed to ensure the successful implementation and long-term sustainability of such interventions.

Given the promise of mHealth interventions, future research should focus on the co-design of gamified interventions rooted in behavior change theories to ensure alignment with their behavioral characteristics, preferences, and lived experiences. These interventions should also be rigorously evaluated for their effectiveness within the Swati context and considered complementary strategies within existing Ministry of Health programs aimed at improving HIV-related outcomes among adolescents.

## Supplementary material

10.2196/74207 Multimedia Appendix 1 The interview guide.

10.2196/74207 Multimedia Appendix 2 Themes and subthemes including exemplary quotes on the use of gamified interventions to enhance antiretroviral therapy adherence among adolescents living with HIV.

## Abbreviations

ART: antiretroviral therapy
CATS: community adherence to treatment support
IDI: in-depth interview
IMB: information-motivation-behavioral
mHealth: mobile health
SNAP: Swaziland National AIDS Program
SSA: sub-Saharan Africa
UNAIDS: Joint United Nations Programme on HIV/AIDS
VLS: viral load suppression

## References

[1] (2023). Eswatini population-based HIV impact assessment 3 2021 (SHIMS3 2021): final report. https://phia.icap.columbia.edu/wp-content/uploads/2023/12/241123_SHIMS_ENG_RR3_Final-1.pdf.

[2] (2022). Eswatini surpasses UNAIDS fast-track targets for treatment and viral suppression. United States Department of State.

[3] (2017). Swaziland HIV incidence measurement survey 2: a population-based HIV impact assessment SHIMS2 2016–2017. http://phia.icap.columbia.edu/wp-content/uploads/2017/11/Swaziland_new.v8.pdf.

[4] Nkambule R, Nuwagaba-Biribonwoha H, Mnisi Z, Ao T Substantial progress in confronting the HIV epidemic in Swaziland: first evidence of national impact [Abstract: MOAX0204LB]. https://www.iasociety.org/sites/default/files/Conference%20reports%202011/IAS2017_conference_report.pdf.

[5] Byrd KK, Hou JG, Hazen R (2019). Antiretroviral adherence level necessary for HIV viral suppression using real-world data. J Acquir Immune Defic Syndr.

[6] Cohen MS, Chen YQ, McCauley M (2016). Antiretroviral therapy for the prevention of HIV-1 transmission. N Engl J Med.

[7] Rodger AJ, Cambiano V, Bruun T (2016). Sexual activity without condoms and risk of HIV transmission in serodifferent couples when the HIV-positive partner is using suppressive antiretroviral therapy. JAMA.

[8] (2023). Global HIV & AIDS statistics—fact sheet. UNAIDS (Joint United Nations Programme on HIV/AIDS).

[9] (2022). Guidelines for differentiated service delivery. https://cquin.icap.columbia.edu/wp-content/uploads/2022/07/Final_Policy-Guidelines-for-Eswatini-Differentiated-Service-Delivery.pdf.

[10] Mbuagbaw L, Sivaramalingam B, Navarro T (2015). Interventions for enhancing adherence to antiretroviral therapy (ART): a systematic review of high quality studies. AIDS Patient Care STDS.

[11] Kharsany ABM, Karim QA (2016). HIV infection and AIDS in sub-Saharan Africa: current status, challenges and opportunities. Open AIDS J.

[12] Hlophe LD, Tamuzi JL, Shumba CS, Nyasulu PS (2023). Barriers and facilitators to anti-retroviral therapy adherence among adolescents aged 10 to 19 years living with HIV in sub-Saharan Africa: a mixed-methods systematic review and meta-analysis. PLoS One.

[13] Munyayi FK, van Wyk B, Mayman Y (2022). Interventions to improve treatment outcomes among adolescents on antiretroviral therapy with unsuppressed viral loads: a systematic review. Int J Environ Res Public Health.

[14] Griffee K, Martin R, Chory A, Vreeman R (2022). A systematic review of digital interventions to improve ART adherence among youth living with HIV in sub-Saharan Africa. AIDS Res Treat.

[15] Okonji EF, Mukumbang FC, Orth Z, Vickerman-Delport SA, Van Wyk B (2020). Psychosocial support interventions for improved adherence and retention in ART care for young people living with HIV (10-24 years): a scoping review. BMC Public Health.

[16] Casale M, Carlqvist A, Cluver L (2019). Recent interventions to improve retention in HIV care and adherence to antiretroviral treatment among adolescents and youth: a systematic review. AIDS Patient Care STDS.

[17] Reif LK, Abrams EJ, Arpadi S (2020). Interventions to improve antiretroviral therapy adherence among adolescents and youth in low- and middle-income countries: a systematic review 2015-2019. AIDS Behav.

[18] Cooper V, Clatworthy J, Whetham J, Consortium E (2017). mHealth interventions to support self-management in HIV: a systematic review. Open AIDS J.

[19] Goldstein M, Archary M, Adong J (2023). Systematic review of mHealth interventions for adolescent and young adult HIV prevention and the adolescent HIV continuum of care in low to middle income countries. AIDS Behav.

[20] Chory A, Callen G, Nyandiko W (2022). A pilot study of a mobile intervention to support mental health and adherence among adolescents living with HIV in western Kenya. AIDS Behav.

[21] (2024). Measuring digital development: facts and figures 2024. https://www.itu.int/itu-d/reports/statistics/wp-content/uploads/sites/5/2024/11/2402588_1e_Measuring-digital-development-Facts-and-Figures-2024_v4.pdf.

[22] (2024). Swaziland Number of Subscriber Mobile. CEIC.

[23] Sahu M, Gandhi S, Sharma MK (2019). Mobile phone addiction among children and adolescents: a systematic review. J Addict Nurs.

[24] Hwang Y, Park N (2017). Is smartphone addiction comparable between adolescents and adults? Examination of the degree of smartphone use, type of smartphone activities, and addiction levels among adolescents and adults. Int Telecommun Policy Rev.

[25] Jeong SH, Kim H, Yum JY, Hwang Y (2016). What type of content are smartphone users addicted to?: SNS vs. games. Comput Human Behav.

[26] Majeed-Ariss R, Baildam E, Campbell M (2015). Apps and adolescents: a systematic review of adolescents’ use of mobile phone and tablet apps that support personal management of their chronic or long-term physical conditions. J Med Internet Res.

[27] Abraham O, LeMay S, Bittner S, Thakur T, Stafford H, Brown R (2020). Investigating serious games that incorporate medication use for patients: systematic literature review. JMIR Serious Games.

[28] Grove SK, Gray JR (2019). Understanding Nursing Research: Building an Evidence-Based Practice.

[29] (2024). Eswatini national development plan 2023/24–2027/28. https://www.gov.sz/images/planningministry/National-Development--Plan-2023-2028.pdf.

[30] (2017). Service availability and readiness assessment 2017. https://data-archive.hhfa.online/index.php/catalog/85/related-materials.

[31] Ahmed CV, Weissinger G, Teitelman A (2022). Expert client service delivery practices among adolescents living with HIV in Eswatini: a thematic analysis. Child Youth Serv Rev.

[32] Creswell JW, Creswell JD (2017). Research Design: Qualitative, Quantitative, and Mixed Methods Approaches.

[33] (2016). National Policy Guidelines FOR Community-Centred Models of ART Service Delivery (CommART) in Swaziland. https://www.differentiatedservicedelivery.org/wp-content/uploads/Swaziland-Policy-Guideline-2016-1-1.pdf.

[34] Audi C, Jahanpour O, Antelman G (2021). Facilitators and barriers to antiretroviral therapy adherence among HIV-positive adolescents living in Tanzania. BMC Public Health.

[35] Kamangu JWN, Mboweni SH (2024). Factors impacting ART adherence among HIV-positive older adolescents and younger adults in Namibia: a qualitative analysis. Open Public Health J.

[36] Bingham AJ (2023). From data management to actionable findings: a five-phase process of qualitative data analysis. Int J Qual Methods.

[37] Cypress BS (2017). Rigor or reliability and validity in qualitative research: perspectives, strategies, reconceptualization, and recommendations. Dimens Crit Care Nurs.

[38] Doyle AM, Bandason T, Dauya E (2021). Mobile phone access and implications for digital health interventions among adolescents and young adults in Zimbabwe: cross-sectional survey. JMIR mHealth uHealth.

[39] (2024). Information and communications technology-sector report. https://www.esccom.org.sz/publications/reports/docs/Esccom%20sector%20report.pdf.

[40] (2025). Eswatini. World Bank Group.

[41] Tarantino N, Lartey M, Arnold T, Brown L, Kwara A, Guthrie K (2022). Preferences for a game-based SMS adherence intervention among young people living with HIV in Ghana: a qualitative study. AIDS Behav.

[42] Fitzgerald M, Ratcliffe G (2020). Serious games, gamification, and serious mental illness: a scoping review. Psychiatr Serv.

[43] Sardi L, Idri A, Fernández-Alemán JL (2017). A systematic review of gamification in e-Health. J Biomed Inform.

[44] Howard C, Bevins K (2018). Game mechanics and why they are employed: what we know about gamification so far. Issues Trends Educ Technol.

[45] Garett R, Young SD (2019). Health care gamification: a study of game mechanics and elements. Tech Know Learn.

[46] Whiteley L, Brown LK, Mena L, Craker L, Arnold T (2018). Enhancing health among youth living with HIV using an iPhone game. AIDS Care.

[47] Ankrah DN, Koster ES, Mantel-Teeuwisse AK, Arhinful DK, Agyepong IA, Lartey M (2016). Facilitators and barriers to antiretroviral therapy adherence among adolescents in Ghana. Patient Prefer Adherence.

[48] Galea JT, Wong M, Muñoz M (2018). Barriers and facilitators to antiretroviral therapy adherence among Peruvian adolescents living with HIV: a qualitative study. PLoS One.

[49] Al-Rayes S, Al Yaqoub FA, Alfayez A (2022). Gaming elements, applications, and challenges of gamification in healthcare. Inform Med Unlocked.

[50] Bezabih AM, Gerling K, Abebe W, Abeele VV (2021). Behavioral theories and motivational features underlying eHealth interventions for adolescent antiretroviral adherence: systematic review. JMIR mHealth uHealth.

[51] Ahonkhai AA, Pierce LJ, Mbugua S (2021). PEERNaija: a gamified mHealth behavioral intervention to improve adherence to antiretroviral treatment among adolescents and young adults in Nigeria. Front Reprod Health.

[52] Munyayi FK, van Wyk B (2020). The effects of teen clubs on retention in HIV care among adolescents in Windhoek, Namibia. South Afr J HIV Med.

[53] Hightow-Weidman L, Muessig K, Knudtson K (2018). A gamified smartphone app to support engagement in care and medication adherence for HIV-positive young men who have sex with men (AllyQuest): development and pilot study. JMIR Public Health Surveill.

[54] Denison JA, Banda H, Dennis AC (2015). “The sky is the limit”: adhering to antiretroviral therapy and HIV self-management from the perspectives of adolescents living with HIV and their adult caregivers. J Int AIDS Soc.

[55] Campbell BR, Choi K, Neils MG (2021). Mobile device usage by gender among high-risk HIV individuals in a rural, resource-limited setting. Telemed J E Health.

[56] Whiteley LB, Olsen EM, Haubrick KK, Odoom E, Tarantino N, Brown LK (2021). A review of interventions to enhance HIV medication adherence. Curr HIV/AIDS Rep.

[57] Côté J, Rouleau G, Ramirez-Garcia MP, Auger P, Thomas R, Leblanc J (2020). Effectiveness of a web-based intervention to support medication adherence among people living with HIV: web-based randomized controlled trial. JMIR Public Health Surveill.

[58] Voorheis P, Zhao A, Kuluski K (2022). Integrating behavioral science and design thinking to develop mobile health interventions: systematic scoping review. JMIR mHealth uHealth.

[59] Amico KR, Mugavero M, Krousel-Wood MA, Bosworth HB, Merlin JS (2018). Advantages to using social-behavioral models of medication adherence in research and practice. J Gen Intern Med.

