# Supplementary material for: Mobile Phone Access, Usage Patterns, and Perceptions of Adolescents Living With HIV on the Use of Gamified Interventions to Improve Antiretroviral Therapy Adherence in Eswatini: Qualitative Study
Source: JMIR Mhealth Uhealth. 2026 Jul 24;14:e74207. doi: 10.2196/74207 (PMC13399411; doi:10.2196/74207)
Supplement: Multimedia Appendix 1 [file mhealth-v14-e74207-s001.pdf]

ART COMPLIANCE EXPERIENCES AND PERCEPTIONS AMONG ADOLESCENTS AGED 10 TO 19 YEARS LIVING WITH HIV AND ON ART IN ESWATINI

1. DEMOGRAPHIC AND PERSONAL CHARACTERISTICS

1.1 How old are you?

……………….

1.2 Sex

Male ☐

Female ☐

1.3 What is the highest level of education you have completed?

…………………………………………………

1.4 How long have you been on ART?

………………………………………………….

1.5 How long have you been a Teen Club member?

……………………………………………………

2. MOBILE PHONE ACCESS

2.1 Do you own or have access to a phone?

Yes, I own a phone ☐

Yes but its shared ☐ Explain (who do you share with) ………………………………….

No ☐ If no, go to 3.

2.2 If yes in 2.1, what type of phone do you own or have access to?

Smart phone ☐

Feature/ basic phone ☐

Any available ☐

2.3 How often do you use a phone?

Everyday ☐

Twice a week ☐

More than twice a week ☐

Weekends only ☐

During school holidays ☐

Other ☐ Explain………………………………………………………………………

2.4 How long do you use a phone each day?

Less than 1 hour per day ☐

1 to 3 hours daily ☐

More than 3 hours daily ☐

Other (explain): ☐ ……………………………………………………………….

2.5 What do you normally use the phone for?

Games ☐

Social media (WhatsApp, Facebook, Instagram etc) ☐ List:………………

Calling ☐

SMSes ☐

Music ☐

Other ☐[Explain]…………………………………………………………………

3. MEDICATION COMPLIANCE EXPERIENCE

Note to interviewer: this is about the ART adherence patterns, facilitators, and barriers to ART adherence.

a. How do you keep up with ensuring you take your medication correctly?

…………………………………………………………………………………………………
…………………………………………………………………………………………………
……………………………………

b. In a week and or month, how often do you miss your medication dose?

……………………………………………………………………………………………
……………………………………………………………….

c. What are the reasons for missing your medication?

……………………………………………………………………………………………
……………………………………………………………………………………………
……………………………………………………………………………………………
…………………………………………………………………………

d. How do you normally overcome missing the medication dose if it has happened before?

……………………………………………………………………………………………
……………………………………………………………………………………………
……………………………………………………………………………………………
………………………………………

4. IMPROVING MEDICATION COMPLIANCE

Note to interviewer: proposed facilitators to ART adherence and use of digital technology to promote ART adherence.

a. What do you think can assist you and other adolescents to take medication well?

……………………………………………………………………………………………
……………………………………………………………………………………………
……………………………………………………………………………………………
……………………………………………………………….

b. Do you believe digital technology especially mobile games can be used to improve ART adherence? If yes, how? Probes

……………………………………………………………………………………………
……………………………………………………………………………………………
……………………………………………………………………………………………
……………………………………

5. Would you be interested in working with us in the design of the mobile gaming application aimed at improving medication compliance?

Yes ☐

No ☐

We have reached the end of our questions but before we conclude the interview, do you have any questions or comments that you would like to share with us? If yes, write them.

……………………………………………………………………………………………
……………………………………………………………………………………………
……………………………………………………………………………………………
……………………………………………

THANK YOU FOR YOUR PARTICIPATION IN THE STUDY

Time taken: ………………………………
