# Supplementary material for: Mobile Phone Access, Usage Patterns, and Perceptions of Adolescents Living With HIV on the Use of Gamified Interventions to Improve Antiretroviral Therapy Adherence in Eswatini: Qualitative Study
Source: JMIR Mhealth Uhealth. 2026 Jul 24;14:e74207. doi: 10.2196/74207 (PMC13399411; doi:10.2196/74207)
Supplement: Multimedia Appendix 2 [file mhealth-v14-e74207-s002.pdf]

Themes and sub-themes including exemplary quotes on the use of gamified interventions to enhance ART adherence among ALHIV

Themes	Sub-themes	Code	Example
**1. Gamified interventions are feasible**	1. Phone access and use	a. Previous game exposure	*"You see auntie, we play lots of games so this can work too but then like myself I can only play it once in a while you see because I don't own the phone… but it can help"* Female ALHIV, aged 15.
		b. Positive game experience	a. *"I play games in my mother's phone like always… I like Talking Angela [a videogame app] and my*

			mom used to play it too (laughs)" Female ALHIV, aged 10.
			b. *"I think it can work auntie especially among the young ones because those ones enjoy playing games… myself, I am not a game person but into blogs and other social media platforms you see"*

			Male ALHIV and peer educator, aged 19.
**2. Qualities of gamified interventions**	1. Supportive	a. Reminders	"*You see auntie, a game where like when you play you learn in the process. Like when you play you can even remember to take your pill and know why it is important.*" Female ALHIV, aged 18.
		b. Support group	*"It would be nice to have a game where you can meet others like a game with multiple players and you are able to support each other with things like taking your pills."* Male ALHIV and peer

			educator, aged 19.
	2. Educational	a. Benefits of ART	*"For me a game where you are fighting using a gun and when you shoot it's an indication that what happens or the results of taking your pills."* Male ALHIV, aged 17.
		b. Living with HIV	*"I think a game that will teach people when they play about life with HIV so that they know more and the things to do to make sure one stays well."* Female ALHIV, aged 14.
	3. Ensure privacy	a. Dependable/ trustworthy	"*You know a game where I can be in my home and play with someone who is in Manzini, that one can work and even better if I*

			know the person is also living with HIV and we can even chat outside the game even though we don't know each other… sometimes talking to a stranger is better auntie because they won't gossip about you" Male ALHIV, aged 19.
		b. Prevention of unintended disclosure	"*A game that does not have a name about HIV can work (laughs), that I can play auntie, and I can enjoy as long as no hint about my status (laughs)*" Female ALHIV, aged 17.
**3. Potential concerns about gamified interventions**	1. Confidentiality	Unintended disclosure	"*A game I can play and even if someone can see but will not be able to tell it's for people living*

			with HIV… like my cousins can also play it without them knowing it's for us" Female ALHIV, aged 14.
	2. Phone access factors	a. Phone costs	"*Mobile games can work auntie, but problem is like myself, I don't have a phone and the one I use is not a smart phone so this will not benefit some of us*" Female ALHIV, aged 15.
		b. Internet bundles cost	"*You see auntie, some of the adolescents here cannot afford to buy internet bundles and you know some games need you to be online"* Male ALHIV, aged 19.

		c. Network availability	"*I don't know auntie, but you know there are places where there is no network… (laughs) you see at my granny's place, there is no network it's just some odd area right… (laughs)*" Female ALHIV, aged 18.
